# Assessing the Efficiency of Molecular Markers for the Species Identification of Gregarines Isolated from the Mealworm and Super Worm Midgut

**DOI:** 10.3390/microorganisms6040119

**Published:** 2018-11-27

**Authors:** Chiara Nocciolini, Claudio Cucini, Chiara Leo, Valeria Francardi, Elena Dreassi, Antonio Carapelli

**Affiliations:** 1 Polo d’Innovazione di Genomica Genetica e Biologia, 53100 Siena, Italy; nocciolinichiara@gmail.com; 2 Department of Life Sciences, University of Siena, 53100 Siena, Italy; claudio.cucini@student.unisi.it (C.C.); leo6@student.unisi.it (C.L.); 3 Consiglio per la Ricerca in agricoltura e analisi dell’Economia Agraria—Centro di Difesa e Certificazione CREA-DC) Research Centre for Plant Protection and Certification, via di Lanciola 12/A, 50125 Firenze, Italy; valeria.francardi@crea.gov.it; 4 Department of Biotechnology, Chemistry and Pharmacy, University of Siena, 53100 Siena, Italy; elena.dreassi@unisi.it

**Keywords:** gregarine, Apicomplexa, species identification, molecular taxonomy, molecular phylogenetics, edible insects

## Abstract

Protozoa, of the taxon Gregarinasina, are a heterogeneous group of Apicomplexa that includes ~1600 species. They are parasites of a large variety of both marine and terrestrial invertebrates, mainly annelids, arthropods and mollusks. Unlike coccidians and heamosporidians, gregarines have not proven to have a negative effect on human welfare; thus, they have been poorly investigated. This study focuses on the molecular identification and phylogeny of the gregarine species found in the midgut of two insect species that are considered as an alternative source of animal proteins for the human diet: the mealworm *Tenebrio molitor*, and the super-worm *Zophobas atratus* (Coleoptera: Tenebrionidae). Gregarine specimens were isolated from the gut of both larval and adult stages of *T. molitor* specimens, as well as from *Z. atratus* larvae. The morphological analyses were restricted to the identification of the different parasite morpho-types, likely corresponding either to different life-cycle stages or to alternative gregarine species. The samples were also used for the DNA extraction necessary for their genetic characterization. Finally, the efficiency of different molecular markers (i.e., *18S* rDNA gene alone or combined with the Internal Transcribed Spacer 1) was assessed when applied either to gregarine species identification and to phylogenetic inference.

## 1. Introduction

According to the Food and Agriculture Organization (FAO) reports, it is widely accepted that by 2050 the human population will amount to about nine billion people and that food production should be almost doubled in order to fill the demand. However, the current methods of growing food are not viable and effective enough to reach this goal. Despite a cultural refusal to introduce them into the everyday diet, insects may represent an eco-sustainable and a highly nutritious food source [1]. The insects’ species broadly employed for food and/or feed use belong mainly to the order Coleoptera, such as the mealworm (*Tenebrio molitor*) (Coleoptera: Tenebrionidae) and the super worm (*Zophobas atratus*) (Coleoptera: Tenebrionidae). In addition to the positive cost/benefit ratio of insects breeding and to a healthy food source that they may represent, insects might also cause less zoonotic infections to humans than mammals and birds, though this aspect should be further investigated [1].

Protozoa are among those microorganisms to be monitored to assess foodstuff safety acceptability [2]; thus, it appears mandatory to improve the knowledge on the most common protozoan biota associated with edible insects to shed light on their taxonomic status and potential risk for human health. It is known that different species of insect orders Coleoptera, Diptera and Orthoptera may be vectors of protozoans dangerous for human health. Among them, *Cryptosporidium parvum*, responsible for the majority of infections in humans, displays several morphological peculiarities that separate these species from other coccidians, and molecular data further suggest its close relationship with the primitive apicomplexan gregarine parasites [3]. Therefore, pathogenic Protozoa species may be present also in edible insects representing a possible health risk, especially for children and immunocompromised subjects [4,5]. Although the Gregarinasina is poorly studied, molecular taxonomic approaches highlighted that some species of this taxon may parasitize a wide range of arthropods [6,7,8,9]. Specimens of *T. molitor* and *Z. atratus* are not an exception and could be parasitized by some species of protozoans belonging to the genus *Gregarina* (Apicomplexa: Eugregarinidae), for which accurate identification need to be performed. It has also been observed that gregarines, often present in the insect gut, may cause reduced host fitness, increase host susceptibility to other diseases and, as studied for the tenebrionid *Tribolium castaneum*, also led to the death of the infected host [10]. For this reason, gregarine species have been proposed as control agents of other pest insects [9]. Accordingly, it could not be excluded that some catabolites produced either by the parasite and/or by the infected host may be dangerous for humans, if these insect species are used as a food source.

The taxonomy and phylogeny of gregarines are still incomplete and difficult to study because light microscopy data generally cannot be used for a reliable classification and that electron microscopy and molecular data are fragmentary [11]. Nevertheless, current gregarines classification mostly relies on light-microscopy studies of trophozoites and on the differences of the life-cycle stages (i.e., absence or presence of merogony) [11,12]. Accordingly, three orders have been so far identified: Archigregarinorida, Neogregarinorida and Eugregarinorida.

For many years, observed differences in the morphology of trophozoites have been considered adequate characters for gregarine diagnosis and description. However, given that some morphological features may vary during their life cycle, due for example to the host species stage [13] or diet [14] and considering that there are not sufficient descriptions for many species, the morphology-based taxonomy alone may be unreliable. For this reason, molecular phylogenetic analyses represent now the most important tool applied for the taxonomy of the group and to reconstruct gregarine evolutionary relationships.

The only molecular marker, used for phylogenetic and molecular taxonomic analyses applied to gregarine systematics, is represented by the ribosomal DNA (rDNA), because so far, no mitochondrial genome has been sequenced from gregarine species. Indeed, the mitochondrial DNA (mtDNA) of Apicomplexa exhibits unique molecular features: sometimes it is linear and organized in concatemer and monomeric linear forms (Coccidia, Hemosporidia and Piroplasmida); in other cases, Apicomplexa, such as *Babesia* and *Theileria*, have very small-sized mitochondrial genomes [15]. Moreover, only two whole-genome sequencing approaches have been applied to analyze the nuclear DNA of gregarines. Therefore, both the identification and the phylogenetic reconstruction of these parasites are complex tasks to be achieved in a conventional way (i.e., using as molecular markers the mitochondrial cytochrome *c* oxidase subunit I (*cox1*) gene or nuclear fragments, such as the microsatellites, usually applied for these purposes).

Generally, rDNA sequences are used for phylogenetic analyses at high taxonomic levels because of the presence of conserved domains, useful for examining ancient evolutionary events. Moreover, the slow rate of base changes allows the synthesis of universal primers for amplifying and sequencing rDNA fragments from many species. Among all the ribosomal genes, that encoding for the small ribosomal subunit (also referred to as *18S* or SSU rDNA) is the most widely applied because of its slow nucleotide substitution rate. While this is true for higher metazoans, it has been shown that the *18S* varies among gregarines with a substitution rate that is 15-times higher than in other eukaryotes [11,16]. For this reason, the SSU rDNA is currently used also for species identification and characterization among gregarine species, as well as for phylogenetic studies, together with the non-functional Internal Transcribed Spacers 1–2 (*ITS1–2*), which show a high degree of variation even between closely related species [8,11].

Part of this work focuses on the different gregarine species found in the midgut of the coleopteran *T. molitor*. In this host, Clopton et al. [17] have identified the presence of four species of gregarines, all attached to the insect’s intestinal epithelium: *Gregarina niphandrodes*, *Gregarina polymorpha*, *Gregarina cuneata* and *Gregarina steini* (Eugregarinorida: Gregarinidae). Furthermore, we apply, to our knowledge for the first time, a molecular taxonomic analysis on the species *Gregarina tibengae* described from the *Z. atratus* midgut by Jahnke [18].

Therefore, our study develops along two main research subjects: (1) the evaluation of the effectiveness of rDNA sequences (i.e., the fragment at 3′-end of the *18S plus* the *ITS1* vs. the fragment between the V4 and V8 regions of *18S* only) for gregarine species delimitation, following two different approaches (referred along the text to as “population genetics” and “single trophozoites” approaches); (2) the identification of the gregarine species that parasite specimens of both *T. molitor* and *Z. atratus*, which should be studied to further investigate the metabolic effects of gregarines on the hosts, alongside with possible side effects for human health when these insects are introduced in our diet.

## 2. Materials and Methods

### 2.1. Insects’ Breeding and Isolation

The insects, whose gut parasites have been analyzed in this study, came from two different breeding stocks. Some specimens of *T. molitor* were initially bred at the Consiglio per la Ricerca in agricoltura e l’analisi dell’Economia Agraria-Difesa e Certificazione (CREA-DC) (Florence, Italy) on a wheat, oat flours and brewer’s yeast-based diet *plus* carrots and subsequently transferred to the Laboratory of Systematic Zoology of the Department of Life Sciences (University of Siena, Siena, Italy). The other group of *T. molitor* and specimens of *Z. atratus* were bred directly at the Department of Life Sciences of the University of Siena. The insects were fed on a vegetarian diet (mainly carrots, salad and bread), at room temperature 25 ± 2 °C, 12L:12D photoperiod and 50/70% relative humidity. The breeding has been constantly monitored for more than one year.

Pools of trophozoites were isolated from the intestine of three single larval instars (L1, L2 and L3) and one adult (A) of *T. molitor* (CREA-DC breeding) and from the midgut of three larvae of *Z. atratus* (Z1, Z2 and Z3); single trophozoites were isolated only from two larvae of *T. molitor* collected either in our and CREA-DC breeding (L4 and L5, respectively), as well as from two larvae of *Z. atratus* (Z4 and Z5) (Table 1). The larval instars L2 and L3 of *T. molitor* were located in the same breeding tank differently from the larval instars L1, L4 and L5, and the adult; whereas all the *Z. atratus* specimens were bred in the same tank (Table 1). The specimens of both coleopteran species were divided in different tanks, based either on the initial breeding stocks (i.e., CREA-DC and University of Siena) and on the life-cycle stage, this latter defined by the length of the larva itself (Table 1). In this way, it was possible to establish whether the gregarine species were specific to a particular larval instar. The gregarines were isolated by pulling out the intestine from the insect’s body and detaching them from the peritrophic membrane of the insects, under a stereomicroscope Wild M3Z Stereo Microscope (Leica, Wetzlar, Germany). Single trophozoites were firstly prepared for morpho-type identification and then for the DNA extraction, whereas pools of trophozoites were directly prepared for DNA extraction.

### 2.2. Morpho-Type Identification

In order to identify the different gregarine morpho-types, single trophozoites isolated from the midgut of both *T. molitor* and *Z. atratus* larvae were rinsed with ethanol 70% *v*/*v* and then collocated in a 1.5 mL tube with 50% *v*/*v* ethanol. Each specimen was photographed under Leica Leitz DMRB light microscope with AxioCam MRc5 digital camera (Zeiss, Goettingen, Germany). A general description of the trophozoites morphology was necessary to support the genetic analyses in order to obtain a reliable molecular characterization of the four different species of gregarine that putatively parasitize *T. molitor* [17], as well as *G. tibengae*, which is expected to parasitize *Z. atratus* [18].

### 2.3. DNA Isolation, Polymerase Chain Reaction (PCR), Cloning and Sequencing

After the isolation, pools of gregarines from each life stage (L1–3, A; Z1–3) have been transferred in a 1.5 mL tube with ethanol 70% *v*/*v*. Total DNA extraction was performed using the kit Wizard^®^ SV Genomic DNA Purification System (Promega, Madison, WI, USA). The final elution in 50 µL was used for PCR amplification through universal primer pairs for a fragment comprising the 3′-end of *18S* and the *ITS1*: forward primer f1300 (5′-TGCATGGCCGTTCTTAGTTG-3′) and reverse primer ITS2- 28S (5′-ATATGCTTAAATTCAGGGGG-3′) [8]. Despite previous work [8], this primer pair was not efficient to selectively amplify the gregarine DNA. The sequences obtained, all attributable to the Apicomplexa group under study, were used to design specific primers for our samples: the forward GPO-rRNA-FW2 (5′-GACTTGTCTGGTTAATTCCGATAA-3′) and the reverse GPO-rRNA-RV (5′-AACAATCAGAATAAAAGAAAAAGG-3′). The PCRs were performed in 25 μL of reaction volume constituted of: 2.5 μL of DNA from each sample, 1.25 μL of both forward and reverse primers (10 mM), 2.5 μL of MgCl_2_ (2.5 mM), 2.5 μL of deoxynucleotides (dNTPs, 10 mM), 5 μL of Green GoTaq Flexi Buffer (Promega), 0.125 μL of GoTaq Flexi DNA polymerase (Promega) (5 u/μL), and 9.875 μL of ddH_2_O. All amplifications were obtained in a GeneAmp^®^ 2700 (Applied Biosystem, Foster City, CA, USA) Thermal Cycler, using the following conditions, for each of the 40 cycles: a denaturation step at 94 °C for 1 min, an annealing step at 50 °C for 1 min and an extension step at 60 °C for 90 s. A preliminary denaturation of the double strand was also performed (at 95 °C for 5 min), as well as a final extension step at 72 °C for 7 min. The PCR products were then purified using the kit Wizard^®^ SV Gel and PCR Clean-Up System (Promega), and their DNA concentration assessed by mean of the device Nanodrop ND1000UV vis (NanoDrop Technologies, Wilmington, DE, USA). To avoid unreliable electropherograms due to the heterogeneity of the initial samples (i.e., the entire pool of gregarines isolated from one single specimen of *T. molitor* and of *Z. atratus*), the amplified fragments were cloned using pGEM^®^-T Easy Vector System (Promega). Samples were sequenced using specific primers for the plasmid: M13F (5′-GTAAAAGGACGGCCAGT-3′) and M13R (5′-CACACAGGAAACACCTATGACCAT-3′) and applying Sanger techniques at the core facility of BioFab Research (Rome, Italy), run on a DNA Analyzer ABI 3730. The resulted electropherograms were manually checked with the software Sequencher 4.4.2 (Gene Codes, Ann Arbor, MI, USA). The final procedure led to the data set applied for the “population genetics” approach. Following the results obtained at the end of this initial step, additional analyses were performed (i.e., “single trophozoites” approach) to increase the amount of analyzed nucleotide data, using a larger portion of the *18S* fragment only. Therefore, we downloaded from GenBank (https://www.ncbi.nlm.nih.gov/genbank/) the *18S* sequences included in the data set of the phylogenetic analysis applied in [19,20]. The sequences were initially aligned using the online tool Clustal Omega (https://www.ebi.ac.uk/Tools/msa/clustalo/) and then the alignment was manually adjusted by mean of the software Mesquite [21,22]. A new couple of universal primers was designed in the conserved regions of the *18S* upstream of the V4 and downstream of the V8 hyper-variable regions of different apicomplexan species: 1-18S-GRF (5′-CGGTAATTCCAGCTCCAAT-3′) and 1222-18S-GRR (5′-TGACTTGCGCTTACTAGGG-3′).

In order to obtain a specific genetic characterization of the morpho-types, identified as aforementioned, we decided to extract the DNA from single trophozoites (as said before, referred to as L4 and L5 for *T. molitor* and to Z4 and Z5 for *Z. atratus*), using the kit Wizard^®^ SV Genomic DNA Purification System (Promega). The final purification elution in 50 µL was used for PCR amplification through primers previously designed (1-18S-GRF and 1222-18S-GRR). The PCRs were performed in 25 μL of reaction volume constituted of: 2.5 μL of DNA from each sample, 1.25 μL of both forward and reverse primers (10 mM), 12.5 μL of GoTaq Long PCR Master Mix (Promega), and 7.5 μL of ddH_2_O. All amplifications were obtained in a GeneAmp^®^ 2700 (Applied Biosystem) Thermal Cycler, using the following conditions, for each of the 35 cycles: a denaturation step at 94 °C for 1 min, an annealing step at 50 °C for 1 min and an extension step at 60 °C for 2 min. The PCR products were, then, purified, quantified and cloned as described above. Ten colonies for each trophozoite were sequenced at the core facility of BioFab Research (Rome, Italy).

Since the first screening of cloned sequences, obtained from the single trophozoites isolated from *Z. atratus* (Z4 and Z5), detected a gap of about 140 bp in length, with respect to the *18S* fragment obtained from gregarines isolated from *T. molitor*, a new forward primer was designed complementary to the portion present in the *18S* of gregarines from L4 and L5, but missing in those from Z4 and Z5. A new PCR was then performed as aforementioned, using a primer pair: the forward ZAT-156-18SF (5′-CCGAATGTTTGTTGATTAGGATG) and the reverse 1222-18S-GRR (5′-TGACTTGCGCTTACTAGGG-3′). The amplification was applied as a test to assess whether: (1) the gregarines isolated from *Z. atratus* were characterized by a shorter *18S* sequence with a gap in the V4 region, or (2) they showed two different *18S* paralogs. The software Visualization Applet for RNA (VARNA) [23] was used to reconstruct the putative secondary structure of the *18S* portion between the II and the III domain, in order to graphically highlight the two variants of the gene. The applied nomenclature follows [24].

### 2.4. Assembling the Data Sets and Evaluating the Genetic Variability

The resulting electropherograms, obtained either from pools of gregarines and from single trophozoites of both *T. molitor* and *Z. atratus*, were manually corrected using the program Sequencher 4.4.2 (Gene Codes). Two data sets were assembled and applied for the: (1) “population genetics” (fragment *18S-ITS1*) and (2) “single trophozoites” (between V4–V8 regions of *18S*) approaches, corresponding to nucleotide data obtained from the pools of gregarines and from single trophozoites, respectively. The sequences, of the two data sets, are deposited in GenBank under the accession numbers: MH974688-MH974743. The alignments were obtained through the online software Clustal Omega (https://www.ebi.ac.uk/Tools/msa/clustalo//) and then manually adjusted and trimmed at the 5′- and 3′-ends by means of the software Mesquite [21]. To identify the haplotypes and their relative frequency, within the “gut populations”, the data sets were also submitted to the online program DNAcollapser (http://users-birc.au.dk/biopv/php/fabox/dnacollapser.php) [25]. Sequences obtained from the trophozoite pools (fragment *18S-ITS1*) were aligned to four outgroup species, available on GenBank (*Gregarina* sp., *G. polymorpha*, *Ascogregarina taiwanensis* and *Ascogregarina* sp.; accession numbers in Table S1) resulting in a 441 bp matrix. Instead, nucleotide data from single trophozoites included all the sequences belonging to the species of the genus *Gregarina* available on GenBank *plus* nine taxa belonging to five Gregarinasina genera, used as outgroups (*Amoebogregarina*, *Ascogregarina*, *Cephaloidophora*, *Heliospora*, and *Leidyana*; accession numbers in Table S2) resulting in a 927 bp matrix. Nucleotide variability was assessed, for both data sets, with the package APE developed under the R environment that calculate the proportion (*p*) of nucleotide sites at which two sequences being compared are different [26].

### 2.5. Molecular Phylogenetic Analyses

The phylogenetic analyses were performed using the single trophozoites data set, either including and excluding the hyper-variable regions of *18S*. In order to test whether the inclusion/exclusion of these regions influences the resulting phylogenetic analyses, the online tool GBlock server [27] was applied. Therefore, one data set resulted in a matrix of 927 bp, and another (with 28% of excluded aligned positions) in a 670 bp matrix. The two data sets were applied to two different phylogenetic analyses, performed using both Maximum likelihood (ML) and Bayesian Inference (BI), as implemented in RAxML 8.2.9 [28] and MrBayes 3.2.2 [29], respectively. The Maximum-likelihood analysis was executed using Randomized Axelerated Maximum Likelihood (RAxML), run on the Cyberinfrastructure for Phylogenetic Research (CIPRES) Science Gateway V. 3.3 [30] under the GTR+I+Γ model with eight categories of discrete gamma distribution. The procedure included 100 independent runs of the ML analysis and 1000 replicates of the multi-parametric bootstrap. The software PartitionFinder 2.1.1 [31] was run in order to define the best model of molecular evolution to be used either on the conserved and on the hyper-variable regions of the *18S*, during the BI analysis. The GTR+I+Γ model was selected for the conserved regions, whereas the GTR+Γ for the variable ones. The models of molecular evolution were then applied to MrBayes 3.2.2 program that operated using the following parameters: four chains (three hot, and one cold) have run for 10^6^ generations, with a sampling frequency of one tree every 1000 iterations, and with the 25% of the tree topologies discarded (burn-in step).

## 3. Results

### 3.1. Population Genetics Approach

A number of 10–20 sequences for each different pool of gregarines were analyzed using as a marker the fragment comprising the 3′-end of *18S* and the *ITS1*. Fifty haplotypes were detected from a total of 67 sequences (11 shared and 39 single haplotypes, Table 2).

The genetic distance analysis was performed using the *18S*-*ITS1* data set between: (1) the sequences obtained from gregarines isolated from the three different larval instars and from the adult of *T. molitor*; (2) gregarines isolated from *Z. atratus* larvae; (3) gregarines isolated from both host species; (4) the sequences obtained in the present study and the selected outgroups (average of observed genetic distances in Table 3).

The *p*-distance values, calculated considering only the sequences obtained in this study, are fairly low, even between samples coming from different breeding tanks (i.e., 1.78% among L1 and L2), as well as among specimens isolated from the two insect hosts (i.e., L1 vs. Z1).

These values may be considered within the limits of an intra-specific variability, with respect to the genetic distance (7.58%) observed between the two congeneric outgroup species, *A. taiwanensis* and *Ascogregarina* sp. This value was arbitrarily applied as a threshold for the inter-specific *p*-distances (Table 3). The genetic distance values obtained comparing our sequences with the outgroups were particularly unexpected (Table 3). Considering that, among all the gregarines parasitizing *T. molitor*, *G. polymorpha* is the most studied species, we would have expected that at least some of the *p*-distance values between our sequences and *G. polymorpha* downloaded from the GenBank database, were within the range of intra-specific variability. Actually, there is about a 33% of genetic divergence among them; a value similar to those obtained comparing the species of the genera *Gregarina* and *Ascogregarina* (range between 29–36%), respectively belonging to the families Gregarinidae and Lecudinidae (e.g., 8 vs. 10 in Table 3).

### 3.2. Single Trophozoites Approach

#### 3.2.1. Morphological Characterization of Trophozoites

**Figure 1:**
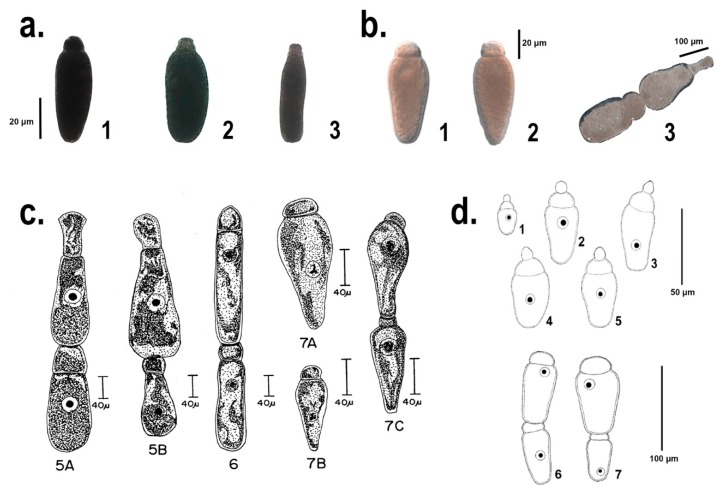
Comparison among the different *Gregarina* species identified and first described in [32] and [18] (**c** 5A–B, 6, 7A–C and **d** 1–7, respectively), and the morpho-type of the single trophozoites, herein identified and characterized from a molecular point of view (**a** 1–3 and **b** 1–3). The section **a**. shows specimens isolated from *T. molitor* midgut, identified following [17] as: 1. *Gregarina polymorpha*, 2. *Gregarina niphandrodes*, 3. *Gregarina cuneata*. The section **b**. shows the single trophozoites and the late association of *Gregarina tibengae* syzygy isolated from *Z. atratus* midgut. Figures from section **c**., reproduced from [32] with permission from John Wiley and Sons, license number 4440151044472, released: 1st of October 2018. Figures from section **d**., reproduced from [18] with permission of the Editor-in-Chief of Acta Protozoologica, Prof. Krzysztof Wiackowski, granted the 1st of October 2018.

Two specimens of *T. molitor* bred at CREA-DC and two bred at the University of Siena, as well as two larvae of *Z. atratus* were dissected. The insects were parasitized by a number of gregarines varying between few individuals up to about 100. We noticed that generally the trophozoites, isolated from *T. molitor*, corresponded to the description of *G. polymorpha* (Figure 1a1; Figure 1c in [17,32]). Few pictures of single trophozoites seemed to have a different morphology. Some specimens had a hemispherical protomerite, globular or conical epimerite without a visible (or barely visible) septum and an elliptical deutomerite, wider through the center, matching the description of *G. niphandrodes* (Figure 1a2; Figure 1a in [17]); some others with a spatula-like protomerite and an elongated and wider posterior deutomerite (Figure 1a3), similar to the description of *G. cuneata* (Figure 1b in [17]). No trophozoites with typical features of *G. steini* were found.

Jahnke [18] described the trophozoites of *G. tibengae*, isolated from the midgut of *Z. atratus* specimens, with a spherical epimerite, a depressed ovoid protomerite and an ovoid deutomerite, with the maximum width at the anterior third (Figure 1d1–5). Moreover, the late association of the syzygy was described as bilaterally symmetric and dorsoventrally flattened ([18]; Figure 1d6–7). However, the trophozoites in the present study, from Z4 and Z5 showed a flattened and elliptical epimerite, an ovoid protomerite and a pear-shaped and widest at the septum deutomerite (Figure 1b1,2). Even the gamonts association, observed in this study, resulted to be fairly different from the one described in [18]: a pear-shape and widest through the posterior primite, a satellite with a hemispherical protomerite and an ovoid deutomerite (Figure 1b3). Unlike from *G. tibengae* [18], our sample, isolated from *Z. atratus*, may be more likely specimens of *G. cuneata* or *G. steini*, if compared with the description in [32] (Figure 1c5A–B, c7A–B).

#### 3.2.2. Molecular Characterization of Trophozoites

**Figure 2:**
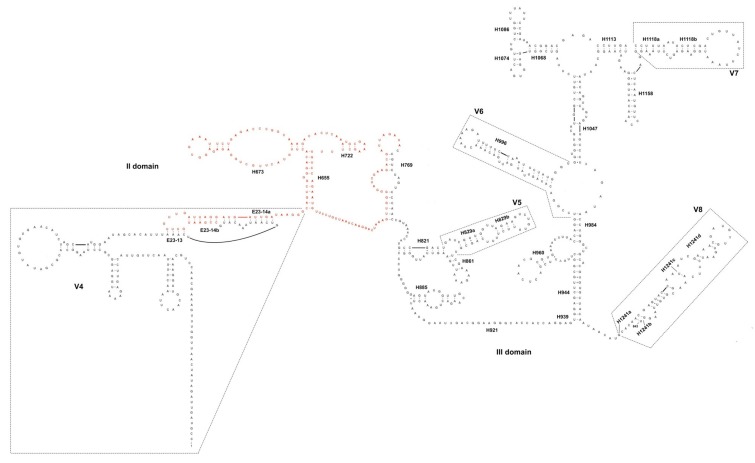
The II and III domains of the *18S* secondary structure of gregarines isolated from *T. molitor* and *Z. atratus* midgut. In red, the portion of the rDNA fragment missing in the gene “variant” detected in *Z. atratus*. Nomenclature of helices and variable regions as in [24].

Due to the complexity to manage so small and delicate samples, not all the single trophozoites were used for the molecular characterization of the gregarines isolated from both *T. molitor* and *Z. atratus* gut. Those specimens that showed different morphology and therefore, could correspond to different gregarine species took precedence. In this case, trophozoites were analyzed using as a nuclear marker a fragment of the *18S* (Figure 2).

Six different haplotypes were identified from a total amount of eight sequences obtained from different trophozoites isolated from L4–5 and Z4–5 (Table 4). The haplotype E and F were shared by two single trophozoites isolated from L5 and the trophozoites isolated from Z4 and Z5, respectively; whereas all the other haplotypes (A–D) were specific for each of the trophozoites analyzed (Table 4).

The application of the almost complete *18S* sequence allowed the inclusion in our analyses of more outgroups, as well as all the sequences available on GenBank, obtained from species belonging to the genus *Gregarina* (accession numbers and taxonomy in Table S2).

The genetic distances were then calculated not only among our sequences, but also between them and: (1) sequences from *Gregarina* species; (2) sequences from the four families: Cephaloidophoridae, Gregarinidae, Lecudinidae and Leidyanidae (Table 5; see also Table S2). The *p*-distance values obtained comparing our sequences (referred to as L4, L5 and Z4 + Z5 in Table 5) resulted to be quite low (minimum and maximum values: 0.27–0.91%, Table 5). Unlike from previous analyses, the application of the *18S* alone to the genetic comparison provided lower estimates. As matter of fact, the sequences obtained from single trophozoites showed levels of genetic distance with *G. polymorpha* coherent with an intra-specific comparison (e.g., 1 vs. 4, Table 5). Low values of *p*-distances (1.44–2.08%) were also observed comparing our sequences with *G. niphandrodes*, and between this latter and *G. polymorpha* (e.g., 1 vs. 5 and 4 vs. 5, Table 5). Speaking at an inter-specific level, *p*-distance values range from 1.6% (e.g., *Gregarina chortiocetes* (7) vs. *Gregarina caledia* (8), Table 5) to about 34% (e.g., *Gregarina cubensis* (19) vs. *G. polymorpha* (4) and vs. *G. niphandrodes* (5), Table 5). Finally, a fairly limited genetic divergence among species has been observed within the outgroup genus *Ascogregarina* (26 vs. 27 and 28; 27 vs. 28, Table 5).

#### 3.2.3. Phylogeny of the Family Gregarinidae

**Figure 3:**
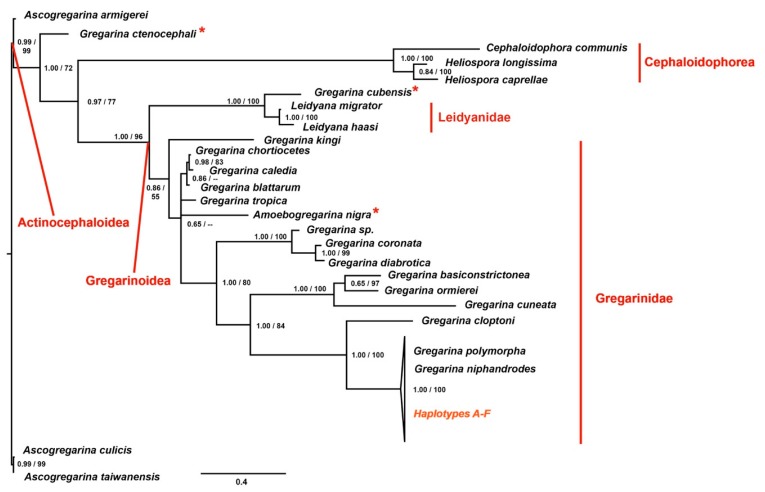
Phylogenetic tree obtained applying to each data set the Bayesian optimization criteria. At each node, the posterior probabilities (alone or before the slash) and the bootstrap values (when the node was shared among the Bayesian Inference (BI) and Maximum likelihood (ML) topologies and when > 50) are shown. Haplotypes A-F are the sequences of the *18S* obtained in the present study. In red, the families and super-families are shown. The asterisks highlight taxa with unexpected grouping within the phylogenetic tree, that lead to para- or polyphyletic lineages.

The phylogenetic analyses were performed using both Maximum likelihood (ML) and Bayesian inference (BI) methods, applied to two different data sets, as described before. The results are shown in Figure 3, in which the BI topology is shown together with the posterior probabilities and the bootstrap values from ML analysis indicated at each node. The trees, obtained using alternative inference criteria, are almost the same (see details afterwards), as well as the node supports (Figure 3). Generally, not all the nodes are fully resolved, and the analyses fail to delimit the reciprocal relationships between the sequences obtained in this study, *G. polymorpha* and *G. niphandrodes*; however, these latter taxa are clustered together in a fully-supported monophyletic lineage. The posterior probabilities are high at almost all nodes of the Bayesian tree, whereas in few cases the bootstrap values were too low to be considered reliable (missing data at some nodes of the tree, in Figure 3).

As expected, the super-family Actinocephaloidea (i.e., the *Ascogregarina* species applied as outgroups) resulted the sister group of all the other species. These latter are distributed into two main clusters: one comprising gregarines belonging to the super-family Gregarinoidea, and the other including gregarines of the super-family Cephaloidophorea. The species *Gregarina ctenocephali* splits from the basal node, as the sister group of the super-families Gregarinoidea and Cephaloidophorea, therefore suggesting the paraphyly of the first taxon (Figure 3). The cluster of the super-family Gregarinoidea splits again into two different branches. One is represented by members of the family Gregarinidae, mainly found inside hosts belonging to Coleoptera (Insecta), Orthoptera (Insecta) and Astigmata (Arachnida). The other is composed by members of the family Leidyanidae *plus G. cubensis*, that should be grouped within Gregarinidae, instead (Figure 3). As expected, our sequences (haplotypes A–F in Figure 3) are grouped together with the species belonging to the family Gregarinasina, and in particular, they are phylogenetically closer to *G. polymorpha* and *G. niphandrodes;* whereas *Gregarina cloptoni* results to be the sister group of these latter taxa.

To sum up, the tree shows some paraphyletic clusters: (1) the super-family Gregarinoidea, since the species *G. ctenocephali* is excluded; (2) the family Gregarinidae, since *G. ctenocephali* and *G. cubensis* are excluded. Furthermore, some of the taxa resulted polyphyletic: (1) the genus *Gregarina*, due to the presence of *Amoebogregarina nigra* within this group; (2) the family Leidyanidae, since *G. cubensis* is included within this taxon (Figure 3).

## 4. Discussion

In order to define the protozoan risk profile of insect-based food, it is necessary, as the first step, to know and characterize the gregarine species that co-exist with the host (e.g., *T. molitor* and *Z. atratus*). Moreover, the definition of a universal method of gregarine species identification is important to extend this research field also to other edible insect species. Therefore, our work tested the efficiency of two different procedures applied to the identification and phylogenetic inference of the species belonging to the genus *Gregarina*.

Given the difficulties linked to the study of gregarine taxonomy, we have deeply investigated, from a molecular point of view, the classification and the phylogeny of those species generally found in the midgut of *T. molitor* [17,32] and of *Z. atratus* [18]. In this respect, we set up two different analyses, referred to as “population genetics” and “single trophozoites” approaches. According to previous works, we would have expected to find: (1) three different species of gregarines (*G. polymorpha*, *G. cuneata* and *G. steini*) within the larvae midgut of *T. molitor*; (2) specimens of *G. niphandrodes* inside the adult gut of *T. molitor*; (3) specimens of *G. tibengae* infecting the larvae of *Z. atratus*.

Following the method suggested in [8], we decided to apply as molecular maker the fragment *18S*-*ITS1*. The Authors suggested that the amplification and sequencing of this fragment would allow a simplified molecular species identification of gregarine taxa, actually proposing the *18S* and both *ITS1-2* as a barcode gene for these apicomplexan group. However, the results obtained in the present study are quite far from the expected. Considering the *p*-distance values among the sequences obtained from the different larval instars of both insect species and the adult of *T. molitor*, it is immediately evident their intra-specific correlation due to the low variability found (0.33–2.61%; Table 3). This data is further supported by the result obtained comparing the genetic distance between two different species belonging to the same genus: *Ascogregarina* sp. and *A. taiwanensis*, which differ each other of about 7% (Table 3). This latter value has been arbitrarily chosen as a threshold, to define the boundaries between the intra- and inter-specific comparison.

Initially, these results have highlighted a discrepancy with the work of Clopton et al. [17], since we would have expected, at least between the adult and the larval stages, a value of variability in the range of different species. Our data further disagreed with previous works, considering the unforeseen results obtained comparing our sequences with those of *Gregarina* sp. and *G. polymorpha* downloaded from GenBank. Moreover, the resulting alignment, inclusive only of a limited portion (at 3′-end) of *18S*, was too short to allow a reliable analysis (i.e., the obtained character matrix was about 441 base pairs in length). However, the divergence observed among our sequences and *Gregarina* sp. and *G. polymorpha* turned out to be alike those obtained between the two species of the genus *Gregarina* and the two other congeneric *Ascogregarina* (about 30%), these latter belonging to two different families: Gregarinidae and Lecudinidae, respectively.

Therefore, the unexpected results described above could be explained by few different hypotheses. First of all, we can suppose, comparing the genetic distances between our sequences with those of *Gregarina* sp. and *G. polymorpha* and between these two latter species, that there is a high degree of variability inside the genus, never detected before. Moreover, we can also suppose that the samples isolated from the larval instars (L1, L2, L3, Z1, Z2 and Z3) and the adults (A) of *T. molitor* and *Z. atratus* could not belong to *G. polymorpha*, but to a different taxon. So far, it is not possible to define if the specimens genetically characterized with this initial approach could be referred to as *G. niphandrodes*, *G. steini*, *G. cuneata* or *G. tibengae*, since they could not be included in the analysis. Looking at these results it may also be possible to hypothesize that our samples could even belong to a different genus or family, selected by the artificial isolation and conditions of massive breeding. Finally, the last hypothesis (and the most parsimonious one) may depend on the molecular markers applied for this study: both the *18S* and *ITS1* are known to be characterized by a higher nucleotide substitution rate in gregarines than in other eukaryotes [8,16]. In particular, the internal transcribed spacers evolve much more rapidly than rDNA genes and accumulate more insertions-deletions, since it has not a functional role during the translation process; thus, being under a more relaxed selective pressure than the ribosomal subunits encoding DNA. Accordingly, our analysis shows that the use of markers, such as the region comprising the 3′-end of the *18S* and the *ITS1*, could not be adequate for both molecular species identification (i.e., it would drastically overestimate the biodiversity) and for inferring the phylogeny of gregarines. Or at least, these markers are not suitable for molecular taxonomy and phylogeny of gregarines that parasitize insect species, but may be efficient if restricted to those parasitizing Arachnida species, as suggested in [8].

In this respect, we decided to make further in-depth analyses, considering only the single trophozoites isolated from both *T. molitor* and *Z. atratus* and comparing molecular data with those obtained from four families of the septate group: Cephaloidophoridae, Gregarinidae, Lecudinidae and Leidyanidae.

According to a general morpho-type observation, most of the trophozoites isolated from *T. molitor* could be correlated with *G. polymorpha*, only two of them to *G. niphandrodes* and *G. cuneata*, respectively (Figure 1a1–3). None of these trophozoites reflected the description of *G. steini*. the identification of a morpho-type isolated from L5, corresponding to the description of *G. niphandrodes* (Figure 1a2)*,* was uncertain especially following [17], in which it is reported that specimens of *G. niphandrodes* should be exclusive of *T. molitor* adults. Among the gregarine speciemens isolated from *Z. atratus*, none of them followed the description of [18] (Figure 1d). Instead, the trophozoites from the super worm midgut were strikingly conformed to the description of *G. steini* (Figure 1b1–2, c7A.B; [32]); whereas the syzygy seemed to reflect that of *G. cuneata* (Figure 1b3, c5A,B; [32]).

The unexpected morphological observation so far described may be explained and discussed analyzing the results obtained from molecular data, which seemed to clarify the anomalies listed above.

The genetic divergence calculated between our sequences and *G. niphandrodes*, as well as between this latter and *G. polymorpha,* showed a very low variability (Table 5), that can be interpreted as an intra-specific comparison; especially, if we consider that the *18S* should vary among gregarines of about 15-times more than in other metazoans [16]. Therefore, according to our results, *G. polymorpha* and *G. niphandrodes* may be synonymous species, as also suggested by [33]. As it often happens in the gregarine’s systematics, the misidentification of these species could be due to the morphological variability of gregarines during the different life-cycle stages. Even though no molecular data were published so far on *G. tibengae*, the *p*-distance values, as well as the phylogeny inferred in this study, would again point for a taxonomic revision of *G. tibengae* too, which should probably be synonymized with *G. polymorpha*. Moreover, it has been confirmed the presence of two *18S* paralogs in gregarines isolated from Z4-5 larvae: one perfectly identical to that of *G. polymorpha*, the other missing a portion of ca 140 bp between the V4 and the conserved helices E23-14b-H769 ([24]; Figure 2). It is not possible to state whether the shorter variant is functional, although the deletion involves a quite long portion of a conserved region (H655-H769, Figure 2). However the presence of a “complete” *18S* sequence, that correspond to *G. polymorpha*, further suggests a taxonomic revision of the gregarine species parasitizing the super worm larvae.

Moreover, the similarity of both the trophozoites and the syzygy of *Z. atratus* with *G. steini* and *G. cuneata*, respectively, is not supported also by a molecular taxonomic approach. As a matter of fact, no conclusion may be stated on the classification of the specimens isolated from *Z. atratus* larvae as belonging to *G. steini* species, since for this species no molecular data are available on public databases. Furthermore, the gregarines found in *Z. atratus* may not be classified as *G. cuneata* since either the *p*-distance calculation and the phylogenetic reconstruction have highlighted a clear evolutionary separation of *G. cuneata* from our sequences, which are instead closer to *G. polymorpha* and *G. niphandrodes* (Table 5; Figure 3). Even the sequence corresponding to the trophozoite isolated from *T. molitor*, which best suited the description of *G. cuneata* (Figure 1a3), showed a variability of about 30% with that downloaded from GenBank, again confirming that gregarine’s morphology is not easy to be described satisfactory [34]. A further aspect that should be taken into account is that, when comparing specimens at low taxonomic levels, general morphology of trophozoites should be interpreted with caution, because some characteristics may be the result of evolutionary convergence, due to the uniformity of host gut environment [11].

Furthermore, this kind of ambiguities in our *p*-distance matrix have been spotted also for other species, showing percentage levels of genetic divergence similar to those observed among *G. polymorpha* and *G. niphandrodes* (e.g., 7 vs. 8 and 9, 8 vs. 9 or 26 vs. 27, in Table 5). On one hand, it is not possible to exclude that values of genetic divergence of approximately 1.2 and 2.5% could be evidence of inter-specific relationships when studying gregarine species. On the other, if we consider the substitution rate of the *18S* reported to be 15-fold higher than in other metazoans [16], and if we decipher those results with parsimony, these low values of genetic divergence should be instead interpreted as the result of an intra-specific comparison.

According to these results, we can conclude that we identified a single species belonging to the genus *Gregarina* isolated either from the midgut of *T. molitor* and from that of *Z. atratus* and that it can be referred to as *G. polymorpha,* rather than *G. niphandrodes*, *G. cuneata* or *G. tibengae.* We can further suppose a positive selection towards *G. polymorpha* species in the different tanks, that might have caused the progressive disappearance of the other species in our breeding, because the former may have better adapted to the artificial environment. Finally, *G. cuneata* is actually a different species, according to genetic distances; therefore, the missed identification in the present study of this species in the midgut of *T. molitor* might be due to a negative selection occurred against *G. cuneata* inside the different breeding tanks, or because it was present in such a limited number of specimens that it was not intercepted by our analyses.

Giving for sure the monophyletic origin of gregarines [11], we focused our attention on some groups belonging to the order Eugregarinorida. Because of so many questions to address for the phylogeny of the Eugregarinorida itself and considering also that our aim was not to focus on the systematics of this order, we have limited our phylogenetic analyses on the family Gregarinidae (see also Table S2).

Following the tree topologies, the taxon Gregarinidae resulted to be paraphyletic, since it includes all the species of the family Gregarinidae, except for *G. ctenocephali* and *G. cubensis*. The sister group of the taxon Gregarinidae includes species of the family Leidyanidae *plus G. cubensis*; accordingly, we can consider this cluster as polyphyletic because it groups members that do not share a more recent common ancestor (Figure 3). This ambiguous classification has been already identified by other works [11,35], in which it has been highlighted the need for a systematic rearrangement of this clade. Indeed, *Leidyana migrator* and *Leidyana haasi* substantially differ from *Leidyana erratica*, which is the type species of the family Leidyanidae. *Leidyana migrator* and *L. haasi* are both described from Blaberidae cockroaches*, Gromphadorina portentosa* and *Neisseria cinerea* respectively, similarly to *G. cubensis*, isolated from another cockroach species, *Blaberus discoidalis*. Considering these characteristics and the phylogenetic distance of *L. migrator* and *L. haasi* with the other members of the family Leidynidae [11,35], it comes to light the need for a new genus description.

Regarding *G. ctenocephali*, which is the first species splitting from the basal node of the tree (Figure 3), we can suppose that also in this case some misidentification could have occurred. As a matter of fact, a different species was isolated from the same host of *G. ctenocephali* (that is *Ctenocephalides steni*) and described as *Steinina ctenocephali*. This latter species belongs to the family Actinocephalidae and to the order Actinocephaloidea [36]. Since *G. ctenocephali* soon splits from the basal node together with the outgroups species, belonging to the super-family Actinocephaloidea, the most parsimonious hypothesis is to consider this species as a synonym of *S. ctenocephali*.

If all the assumptions made regarding both *G. cubensis* and *G. ctenocephali* would be confirmed by morphological and further molecular analyses, the taxa Gregarinoidea, Gregarinidae and *Gregarina* could be considered monophyletic.

Observing the cluster of the genus *Gregarina*, we can distinguish four clades in which are grouped gregarines that parasite different arthropod hosts (Figure 3). This classification is further supported by morphological analyses, since *Gregarina basiconstrictonea* and *G. cuneata* differ from the other gregarine species in overall size, deutomerite morphology and oocysts size and morphology [35]. In addition, *Gregarina diabrotica* and *Gregarina coronata* are characterized by a persistent epimerite until syzygy and by a spatula-like protomerite [35]. Our sequences are grouped together in a collapsed cluster with *G. niphandrodes* and *G. polymorpha*, further confirming the putative synonymy of these species (Figure 3). *G. cloptoni* results to be their sister group as previously highlighted [11,35] and confirmed by the genetic distance value of about 20% (Figure 3; Table 5). Moreover, since *G. cuneata* belongs to an interior clade with *G. basiconstrictionea* and *Gregarina ormierei*, parasites of two different Tenebrionidae—e.g., *T. castaneum* and *Scaurus punctatus*, respectively-the phylogeny suggests that *T. molitor* larvae acquired *G. cuneata* ectopically due to common habitat, rather than through speciation events subsequent to host diversification.

## 5. Conclusions

To sum up, what stands out from this study is first of all the importance of enhancing all the methodologies applied to both species’ identification and phylogeny of the taxon Gregarinasina. This aim could be achieved enlarging the data on gregarine’s genomes (nuclear, mitochondrial and, when present, also the apicoplast’s one). It is also evident the need to define a single molecular marker more suitable for taxon delineation, since it is clear that the fragment 3′-end of *18S plus* the *ITS1* does not sufficiently support gregarine taxonomy and species identification, due to their relaxed selective pressure. However, the *18S* alone, due to the larger number of sequenced taxa and its conserved rate of nucleotide substitution, is still the only gene efficiently used for phylogenetic purpose and species identification in gregarines. Moreover, improving these techniques may be fundamental to lay the foundation for further transcriptomic and biochemical analyses that may shed a light on the effects of gregarine infection in hosts (such as *T. molitor* or *Z. atratus*) candidates to be introduced in the human diet. Since it is not clear whether gregarines should be considered parasites or harmless symbionts, this topic deserves further in-depth analyses, with the aim to establish standard quality assays for the insects’ species introduced in our diet.

## Figures and Tables

**Table 1:** List of the insect specimens applied for both the “population genetics” and the “single trophozoites” approach. The place and the tank in which the coleopteran species were bred are also shown.

Label	Species	Breeding	Tank	Larval Instar Length (cm)
L1	*Tenebrio molitor*	CREA-DC Florence	1	0.9–1.0
L2	*Tenebrio molitor*	CREA-DC Florence	2	1.5–2.5
L3	*Tenebrio molitor*	CREA-DC Florence	2	1.5–2.5
A	*Tenebrio molitor*	CREA-DC Florence	3	-
L4	*Tenebrio molitor*	CREA-DC Florence	4	1.2–1.5
L5	*Tenebrio molitor*	University of Siena	5	2.0–2.5
Z1	*Zophobas atratus*	University of Siena	6	3.0
Z2	*Zophobas atratus*	University of Siena	6	3.1
Z3	*Zophobas atratus*	University of Siena	6	3.2
Z4	*Zophobas atratus*	University of Siena	6	3.3
Z5	*Zophobas atratus*	University of Siena	6	3.4

**Table 2:** List of the haplotypes identified in the “population genetics” approach, with respective label, host and frequency within the insect gut.

Haplotype Label	Host	Haplotype Label	Host
h1	L1	h26	A(2)
h2	L1	h27	A(3), Z3
h3	L1	h28	A
h4	L1	h29	A
h5	L1, L2 (5)	h30	A
h6	L1	h31	A
h7	L1	h32	Z1
h8	L1	h33	Z1
h9	L1	h34	Z1
h10	L1	h35	Z1
h11	L2, Z1	h36	Z1
h12	L2	h37	Z1, Z2
h13	L2	h38	Z1
h14	L2, Z2	h39	Z1
h15	L2	h40	Z2
h16	L3, A	h41	Z2
h17	L3	h42	Z2
h18	L3, Z2	h43	Z2
h19	L3	h44	Z2
h20	L3	h45	Z2
h21	L3, Z1	h46	Z3
h22	L3, Z2	h47	Z3
h23	L3	h48	Z3(2)
h24	L3	h49	Z3
h25	L3	h50	Z3

**Table 3:** The average percentage of *p*-distances calculated applying a “population genetics approach”. Divergence values are assessed: within *Gregarina* specimens belonging to the same midgut micro-community (e.g., L1 vs. L1); among specimens isolated from different stages of *Tenebrio molitor* (e.g., A vs. L1); between different coleopteran species (e.g., L1 vs. Z1 in larvae of *T. molitor* and *Zophobas atratus*, respectively); and between our sequences (number 1 to 7) and those downloaded from GenBank (8-11).

		1	2	3	4	5	6	7	8	9	10	11
**1**	L1	1.60										
**2**	L2	1.78	0.33									
**3**	L3	1.81	2.53	1.53								
**4**	A	1.55	2.02	1.94	1.56							
**5**	Z1	2.29	2.61	1.89	2.63	1.75						
**6**	Z2	2.09	2.53	1.78	2.28	1.93	2.27					
**7**	Z3	1.97	2.27	1.83	2.10	2.16	2.15	1.91				
**8**	*Gregarina polymorpha*	33.25	33.33	33.46	33.15	33.67	33.41	33.11	-			
**9**	*Gregarina* sp.	28.93	29.34	28.85	28.66	29.76	29.30	29.24	32.27	-		
**10**	*Ascogregarina* sp.	34.61	34.47	34.88	34.57	34.35	35.09	34.62	35.94	30.56	-	
**11**	*Ascogregarina taiwanensis*	34.01	33.99	34.15	34.08	34.58	34.35	34.03	33.50	31.05	7.58	-

**Table 4:** List of the haplotypes identified in the “single trophozoites” approach, with respective label, host and frequency within the same insect gut.

Haplotype Label	Host
A	L4(1)
B	L4(1)
C	L4(1)
D	L5(1)
E	L5(2)
F	Z4(1), Z5(1)

**Table 5:** Average *p*-distance values calculated when applying a “single trophozoites” (*18S* only) approach. The divergence values are assessed: between the sequences obtained in this study; among our sequences and all the *Gregarina* spp. for which almost the entire *18S* was available on GenBank; among sequences of *Gregarina* spp. and five outgroup genera (number 20–28).

		1	2	3	4	5	6	7	8	9	10	11	12	13	14	15	16	17	18	19	20	21	22	23	24	25	26	27	28
**1**	L4	0.53																											
**2**	L5	0.27	-																										
**3**	Z4 + Z5	0.91	0.64	-																									
**4**	*Gregarina polymorpha*	0.27	0.00	0.64	-																								
**5**	*Gregarina niphandrodes*	1.71	1.44	2.08	1.44	-																							
**6**	*Gregarina* sp.	29.75	29.49	29.97	29.49	30.29	-																						
**7**	*Gregarina chortiocetes*	28.47	28.21	28.53	28.21	28.85	22.76	-																					
**8**	*Gregarina caledia*	29.27	29.01	29.33	29.01	29.65	23.40	1.60	-																				
**9**	*Gregarina blattarum*	28.47	28.21	28.53	28.21	28.85	22.44	1.76	2.56	-																			
**10**	*Gregarina tropica*	27.83	27.56	27.88	27.56	28.04	20.67	5.77	6.57	5.29	-																		
**11**	*Gregarina kingi*	29.75	29.49	29.81	29.49	29.97	23.40	18.43	18.43	17.63	17.79	-																	
**12**	*Gregarina coronata*	29.59	29.33	29.81	29.33	30.29	8.65	23.08	22.92	22.92	21.63	23.72	-																
**13**	*Gregarina diabrotica*	28.95	28.69	29.17	28.69	29.49	9.13	24.52	24.36	24.52	22.60	23.56	5.45	-															
**14**	*Gregarina cloptoni*	20.46	20.19	20.51	20.19	21.15	29.65	28.37	28.21	28.04	27.24	29.33	29.81	30.77	-														
**15**	*Gregarina basiconstrictonea*	29.59	29.33	29.81	29.33	29.97	25.16	26.12	26.60	25.64	25.16	27.24	25.80	26.28	30.13	-													
**16**	*Gregarina cuneata*	29.65	29.49	30.13	29.49	30.45	27.72	31.57	32.05	31.09	30.93	31.57	29.49	29.01	32.37	22.44	-												
**17**	*Gregarina ctenocephali*	32.05	31.89	32.37	31.89	32.21	29.33	22.44	22.60	22.12	21.63	25.00	29.81	30.61	30.93	29.01	32.37	-											
**18**	*Gregarina ormierei*	28.15	27.88	28.53	27.88	28.53	26.76	25.80	25.80	25.48	25.16	27.08	26.92	28.53	30.77	14.10	22.60	30.77	-										
**19**	*Gregarina cubensis*	34.24	33.97	34.29	33.97	34.13	29.17	23.24	23.24	22.76	23.56	25.64	29.17	29.01	33.17	30.77	33.33	27.72	30.61	-									
**20**	*Amoebogregarina nigra*	28.79	28.53	29.17	28.53	29.17	22.76	14.42	14.42	14.10	15.06	22.28	24.20	25.16	29.81	26.44	30.61	26.12	26.12	26.12	-								
**21**	*Leidyana migrator*	33.28	33.01	33.33	33.01	33.65	27.88	21.31	20.99	20.83	21.79	22.76	29.17	28.85	31.09	29.01	31.73	26.76	28.04	11.86	24.36	-							
**22**	*Leidyana haasi*	33.12	32.85	33.17	32.85	33.49	27.72	20.83	20.51	20.51	21.47	22.76	28.69	29.01	30.13	28.69	32.53	26.60	28.53	12.02	24.68	2.24	-						
**23**	*Cephaloidophora communis*	37.50	37.34	37.82	37.34	37.82	36.22	35.74	36.54	35.58	35.26	36.54	35.58	36.70	37.98	36.86	38.94	33.81	36.54	37.98	36.06	36.54	36.86	-					
**24**	*Heliospora longissima*	39.42	39.26	39.74	39.26	39.58	34.46	36.22	36.70	35.90	34.62	34.62	34.62	35.74	38.14	35.42	36.54	33.17	36.70	36.22	36.70	35.74	36.22	17.47	-				
**25**	*Heliospora caprellae*	38.62	38.46	38.94	38.46	39.10	34.94	34.78	35.58	35.26	33.97	34.78	33.97	35.10	38.14	34.78	35.10	32.05	36.54	36.38	35.26	36.22	35.58	18.27	8.65	-			
**26**	*Ascogregarina armigerei*	31.89	31.73	32.21	31.73	32.53	27.56	22.28	22.28	22.12	21.79	24.84	28.04	28.37	30.45	29.17	31.57	11.86	31.25	28.69	26.12	26.92	26.92	33.01	31.73	32.85	-		
**27**	*Ascogregarina culicis*	31.73	31.57	32.05	31.57	32.37	27.88	22.60	22.60	22.44	22.60	25.16	28.21	28.53	31.09	30.13	32.05	12.18	31.41	29.01	26.28	26.92	26.92	33.49	32.37	33.17	1.12	-	
**28**	*Ascogregarina taiwanensis*	31.57	31.41	31.89	31.41	32.21	27.72	22.44	22.44	22.28	22.28	25.00	28.04	28.37	30.77	29.81	31.89	11.86	31.41	28.85	26.28	26.92	26.92	33.17	32.05	33.01	0.80	0.32	-

## Supplementary Materials

Supplementary materials can be found at http://www.mdpi.com/2076-2607/6/4/119/s1.
