# Supplementary material for: Assessing the Efficiency of Molecular Markers for the Species Identification of Gregarines Isolated from the Mealworm and Super Worm Midgut
Source: Microorganisms. 2018 Nov 27;6(4):119. doi: 10.3390/microorganisms6040119 (PMC6313518; doi:10.3390/microorganisms6040119)
Supplement: Supplementary file 1 [file microorganisms-06-00119-s001.zip › Table S1.docx]

| **Species** | **Family** | **Order** | **Accession number** |
| --- | --- | --- | --- |
| *Gregarina polymorpha* | Gregarinidae | Eugregarinorida | JF459748 |
| *Gregarina sp.* | Gregarinidae | Eugregarinorida | JF412715 |
| *Ascogregarina sp.* | Lecudinidae | Eugregarinorida | KY471625 |
| *Ascogregarina taiwanensis* | Lecudinidae | Eugregarinorida | EF666482 |
