# Supplementary material for: Assessing the Efficiency of Molecular Markers for the Species Identification of Gregarines Isolated from the Mealworm and Super Worm Midgut
Source: Microorganisms. 2018 Nov 27;6(4):119. doi: 10.3390/microorganisms6040119 (PMC6313518; doi:10.3390/microorganisms6040119)
Supplement: Supplementary file 1 [file microorganisms-06-00119-s001.zip › Table S2.docx]

| **Species** | **Family** | **Super-family** | **Order** | **Accession number** |
| --- | --- | --- | --- | --- |
| *Gregarina polymorpha* | Gregarinidae | Gregarinoidea | Eugregarinorida | FJ459748 |
| *Gregarina niphandrodes* | Gregarinidae | Gregarinoidea | Eugregarinorida | DQ837379 |
| *Gregarina sp.* | Gregarinidae | Gregarinoidea | Eugregarinorida | JF412715 |
| *Gregarina chortiocetes* | Gregarinidae | Gregarinoidea | Eugregarinorida | L31841 |
| *Gregarina caledia* | Gregarinidae | Gregarinoidea | Eugregarinorida | L31799 |
| *Gregarina blattarum* | Gregarinidae | Gregarinoidea | Eugregarinorida | FJ459743 |
| *Gregarina tropica* | Gregarinidae | Gregarinoidea | Eugregarinorida | FJ459749 |
| *Gregarina kingi* | Gregarinidae | Gregarinoidea | Eugregarinorida | FJ459746 |
| *Gregarina coronata* | Gregarinidae | Gregarinoidea | Eugregarinorida | FJ459743 |
| *Gregarina diabrotica* | Gregarinidae | Gregarinoidea | Eugregarinorida | FJ459745 |
| *Gregarina cloptoni* | Gregarinidae | Gregarinoidea | Eugregarinorida | FJ459742 |
| *Gregarina basiconstrictonea* | Gregarinidae | Gregarinoidea | Eugregarinorida | FJ459740 |
| *Gregarina cuneata* | Gregarinidae | Gregarinoidea | Eugregarinorida | FJ459744 |
| *Gregarina ctenocephali* | Gregarinidae | Gregarinoidea | Eugregarinorida | GU320208 |
| *Gregarina ormierei* | Gregarinidae | Gregarinoidea | Eugregarinorida | KJ736741 |
| *Gregarina cubensis* | Gregarinidae | Gregarinoidea | Eugregarinorida | FJ459751 |
| *Amoebogregarina nigra* | Gregarinidae | Gregarinoidea | Eugregarinorida | FJ459737 |
| *Leidyana migrator* | Leidyanidae | Gregarinoidea | Eugregarinorida | AF457130 |
| *Leidyana haasi* | Leidyanidae | Gregarinoidea | Eugregarinorida | FJ459753 |
| *Cephaloidophora communis* | Cephaloidophoridae | Cephaloidophorea | Eugregarinorida | HQ891113.2 |
| *Heliospora longissima* | Uradiophoridae | Cephaloidophorea | Eugregarinorida | HQ891115.2 |
| *Heliospora caprellae* | Uradiophoridae | Cephaloidophorea | Eugregarinorida | HQ876007 |
| *Ascogregarina armigerei* | Lecudinidae | Actinocephaloidea | Eugregarinorida | DQ462459 |
| *Ascogregarina culicis* | Lecudinidae | Actinocephaloidea | Eugregarinorida | DQ462457 |
| *Ascogregarina taiwanensis* | Lecudinidae | Actinocephaloidea | Eugregarinorida | DQ462455 |
